# Ablation of NG2 Proteoglycan Leads to Deficits in Brown Fat Function and to Adult Onset Obesity

**DOI:** 10.1371/journal.pone.0030637

**Published:** 2012-01-25

**Authors:** Yunchao Chang, Zhi-Gang She, Kenji Sakimura, Amanda Roberts, Karolina Kucharova, David H. Rowitch, William B. Stallcup

**Affiliations:** 1 Cancer Center, Sanford-Burnham Medical Research Institute, La Jolla, California, United States of America; 2 Department of Cellular Neurobiology, Brain Research Institute, Niigata University, Niigata, Japan; 3 Molecular and Integrative Neurosciences Department, The Scripps Research Institute, La Jolla, California, United States of America; 4 Department of Pediatric Oncology, University of California San Francisco, San Francisco, California, United States of America; Pennington Biomedical Research Center, United States of America

## Abstract

Obesity is a major health problem worldwide. We are studying the causes and effects of obesity in C57Bl/6 mice following genetic ablation of NG2, a chondroitin sulfate proteoglycan widely expressed in progenitor cells and also in adipocytes. Although global NG2 ablation delays early postnatal adipogenesis in mouse skin, adult NG2 null mice are paradoxically heavier than wild-type mice, exhibiting larger white fat deposits. This adult onset obesity is not due to NG2-dependent effects on CNS function, since specific ablation of NG2 in oligodendrocyte progenitors yields the opposite phenotype; i.e. abnormally lean mice. Metabolic analysis reveals that, while activity and food intake are unchanged in global NG2 null mice, O_2_ consumption and CO_2_ production are decreased, suggesting a decrease in energy expenditure. Since brown fat plays important roles in regulating energy expenditure, we have investigated brown fat function via cold challenge and high fat diet feeding, both of which induce the adaptive thermogenesis that normally occurs in brown fat. In both tests, body temperatures in NG2 null mice are reduced compared to wild-type mice, indicating a deficit in brown fat function in the absence of NG2. In addition, adipogenesis in NG2 null brown pre-adipocytes is dramatically impaired compared to wild-type counterparts. Moreover, mRNA levels for PR domain containing 16 (PRDM16) and peroxisome proliferator-activated receptor γ coactivator (PGC)1-α, proteins important for brown adipocyte differentiation, are decreased in NG2 null brown fat deposits i*n vivo* and NG2 null brown pre-adipocytes *in vitro*. Altogether, these results indicate that brown fat dysfunction in NG2 null mice results from deficits in the recruitment and/or development of brown pre-adipocytes. As a consequence, obesity in NG2 null mice may occur due to disruptions in brown fat-dependent energy homeostasis, with resulting effects on lipid storage in white adipocytes.

## Introduction

Obesity is increasingly cited as a worldwide health issue with major medical and economic consequences [1], [2]. Since body weight and composition are largely the result of a balance between energy storage and energy expenditure, obesity represents an imbalance in energy homeostasis. Two different types of adipose tissue, white adipose tissue (WAT) and brown adipose tissue (BAT), play key roles in energy homeostasis. Despite several similarities between white and brown adipocytes, the two cell types arise from different origins. BAT shares a common progenitor with skeletal muscle, with the transcriptional activators PRDM16 and PGC1-α promoting the generation of brown adipocytes [3], [4], [5]. BAT and WAT also differ functionally. Whereas WAT is specialized for energy storage in the form of fat, the major function of BAT is in energy expenditure through heat production in response to either cold or excess calorie intake (adaptive thermogenesis; [6]), providing protective benefits for mammals in cold environments and with variable diets. BAT is highly vascularized, as well as innervated by sympathetic nerves, both of which are important factors in regulating thermogenesis [7]. When animals are exposed to the cold, efferent sympathetic nerves are activated. The resulting release of norepinephrine activates β3-adrenergic receptors (AR) in the plasma membrane of brown fat cells, leading to adenylate cyclase-mediated production of cAMP. Stimulated by cAMP, protein kinase A (PKA) triggers lipolysis and activation of uncoupling protein 1 (UCP1) to uncouple the respiratory chain from ATP production to produce heat [6]. Deficits in brown fat function can affect efficient utilization of energy, resulting in accumulation of high levels of lipids that are then stored in WAT [8], [9]. Although known to be an important aspect of metabolism in small rodents, BAT in larger mammals was previously thought to be important only in newborns [10]. Recent experimental results demonstrate the presence of BAT in humans, and provide evidence for possible interconversion between WAT and BAT [11], [12], [13], [14], [15]. However, BAT involvement in regulating body weight in rodents and humans remains a topic of controversy. Thus, understanding factors that regulate the development and function of BAT is of great importance in the field of metabolic diseases.

Due to the complexity of energy homeostasis, effects on body composition and obesity can arise from unexpected origins. We have been surprised to find that C57Bl/6 mice develop adult-onset obesity as a result of genetic ablation of the NG2 proteoglycan. Also known as the melanoma proteoglycan or CSPG-4, NG2 is expressed by a number of different immature or progenitor cell types, including oligodendrocyte progenitors [16], [17], chondroblasts and osteoblasts [18], [19], immature keratinocytes [20], smooth muscle [21], [22], [23], and microvascular pericytes [22], [24]. NG2 contributes to the proliferation and motility of these cells prior to maturation, and is normally down-regulated as they undergo terminal differentiation [25]. In all cases examined so far *in vivo*, including pericytes [26], [27], oligodendrocyte progenitors [28], keratinocytes [20] and white adipocytes in the subcutis layer of the early postnatal skin [20], ablation of NG2 is associated with deficits in progenitor cell development and the sizes of progenitor pools. Thus, it was unexpected to find that adult NG2 null mice become obese and develop several obesity-associated traits. This communication describes NG2-dependent deficits in BAT that underlie the development of this obese phenotype.

## Materials and Methods

All experimental work was carried out according to Office of Laboratory Animal Welfare guidelines, subsequent to approval by the Sanford-Burnham Institutional Animal Care and Use Committee.

### Animals

Wild-type and NG2 null C57Bl/6 mice [29] were maintained as separate colonies in the Association for Assessment and Accreditation of Laboratory Animal Care-accredited Sanford-Burnham vivarium. For cold challenge testing, three-month old male mice were caged individually and subjected to cold exposure (4°C) with free access to food and water. Mouse body temperatures were measured using a TH-5 Thermalert thermometer (Physitemp, Clifton, NJ) with rectal probe. The high-fat diet used in this study is Teklad Adjusted Calories Western-type diet (21% fat, 19.5% casein, and 0.15% cholesterol, TD 88137).

### Generation of Oligodendrocyte Progenitor-specific NG2 null mice

To produce mice with specific ablation of NG2 in oligodendrocyte progenitor cells (OPCs), we crossed NG2 floxed mice with Olig2-Cre transgenic mice. For generating the NG2 floxed mice, a genomic fragment of the NG2 gene (*Cspg4*) was isolated from a C57Bl/6 mouse genomic BAC clone obtained from BACPAC Resources Center (Oakland, CA). A 1.8-kb DNA fragment carrying the 34-bp loxP sequence and a neo cassette flanked by two frt sites was inserted into the NG2 sequence at a site 192 bp upstream of exon 2 (exon 2 is 164 bp in length). A second 34-bp loxP sequence was inserted at a site 149 bp downstream of exon 2. The targeting vector ptvNG2-flox thus contained exon 2 of the NG2 gene flanked by loxP sequences, 6.6 kb upstream and 5.2 kb downstream genomic sequences, and 4.3 kb pDEST. The construct was designed so that Cre-mediated recombination not only excises exon 2, but also introduces a frameshift mutation that disrupts coding for the downstream portion of NG2. The targeting vector was linearized and electroporated into the C57Bl/6N ES cell line RENKA. G418-resistant clones were selected and analyzed via Southern blot analysis. Correctly-targeted clones were injected into eight-cell stage CD-1 mouse embryos, which were cultured to produce blastocysts and then transferred to pseudo pregnant CD-1 females. Resulting male chimeric mice were crossed with female C57BL/6N mice to establish the NG2 floxed line. Probes for Southern blotting were as follows. 5′ probe forward: 5′-GAC ACA GTG GGA AGG GAG AT-3′, 5′ probe reverse: 5′-TAT CAT ACT GGC CAG TGC CC-3′; 3′ probe forward: 5′-TTG ACT AGG TGG TCC AGG AG-3′, 3′ probe reverse: 5′-GAA TGA TCC TCC TTC TGC AG-3′.

The Olig2-Cre transgenic mouse has been previously described [30]. Olig2 is expressed specifically in cells of the oligodendrocyte lineage and in a small subpopulation of cerebellar granule neurons. Since NG2 is expressed by OPCs and not by granule neurons, the Olig2-Cre mouse is perfectly suited for ablating NG2 specifically in OPCs.

### Histology

Epididymal WAT pads and interscapular BAT were fixed with 4% paraformaldehyde overnight and cryoprotected in 20% sucrose. Cryosections (10–20 µm) were stained with hematoxylin and eosin (Sigma-Aldrich, St. Louis, MO) or Oil Red O. In vitro differentiated cells were fixed with buffered 4% paraformaldehyde for 20 min and stained with Oil red O (0.5% in 60% isopropanol) for 10 min. In some experiments, the relative intensity of Oil red O signal was quantified by ImagePro software (Diagnostic Instruments, USA).

### Immunofluorescence

Immunofluorescence analysis was performed on cryosections or cells fixed with buffered 4% paraformaldehyde as described previously [28] using the following antibodies: rabbit anti-PDGFRβ [27], rabbit anti-PDGFRα [28], guinea pig anti-NG2 [28], rat anti-CD31 (BD Pharmingen, La Jolla, CA) and Cy3 or Cy2-conjugated second antibodies (Jackson ImmunoResearch, PA). Pre-incubation of adipose tissue sections in 0.1% Sudan Black B in 70% Ethanol was used to reduce autofluorescence. Images were captured by Fluoview 1000 Olympus Laser Point Scanning Confocal Microscope and processed by Image J software.

### Physiological Metabolic Tests

Tests were done in the Mouse Animal Models Core, The Scripps Research Institute (La Jolla, CA). Indirect calorimetry was performed on acclimated, singly-housed mice using a computer-controlled, open-circuit system (Oxymax System) that was part of an integrated Comprehensive Lab Animal Monitoring System (CLAMS; Columbus Instruments, Columbis, OH). Testing occurred in clear respiratory chambers (20×10×12.5 cm) equipped with a sipper tube delivering water, food tray connected to a balance, and 16 photobeams situated in rows at 0.5 in intervals to detect motor activity along the x-and z-axes. Room air was passed through chambers at a flow rate of 0.5 L/Min. Exhaust air from each chamber was sampled at 15-min intervals for 1 min. Sample air was sequentially passed through O_2_ and CO_2_ sensors (Columbus Instruments) for determination of O_2_ and CO_2_ content, from which measures of O_2_ consumption (V O_2_) and CO_2_ production (VCO_2_) were estimated. Outdoor air reference values were sampled after every 8 measurements. Gas sensors were calibrated prior to the onset of experiments with primary gas standards containing known concentrations of O_2_, CO_2_, and N_2_ (Airgas Puritan Medical, Ontario, CA). Mice undergoing indirect calorimetry were acclimated to the respiratory chambers for 3–4 days before the onset of study. Data were recorded at ambient room temperature (∼24–26°C) for 24 hours up to 7 days. Mice were health checked daily and food and water changed at least twice a week.

### Adipogenesis Assays

Murine embryonic fibroblasts (MEFs) were obtained from embryos at 13.5 days of gestation. Embryos were dissected from pregnant mice, cleared of head and red organs, minced and digested in DMEM containing type I collagenase (1 mg/mL) at 37°C with frequent shaking for 30 minutes. Digested tissues were filtered through sterile 70 µm nylon mesh and centrifuged at 250 *g* for 5 minutes. The pellets were then re-suspended in DMEM containing 10% FBS and grown at 37°C in 5% CO2. Early passages (less than 5) of MEFs were used for the adipogenesis assay.

Interscapular brown adipose tissue was dissected from newborn mice, minced and digested in DMEM containing type I collagenase (1 µmg/mL) at 37°C with frequent shaking for 30 minutes. Digested tissues were filtered through sterile 70 µm nylon mesh and centrifuged at 250 *g* for 5 min The pellets representing the stromo-vascular fractions were then re-suspended in MSC expansion medium (SCM015, Millipore, CA) and grown at 37°C, 5% CO2. Primary brown pre-adipocytes (less than passage 4) were immortalized by SV40 virus infection [31], [32] and resulting colonies were pooled for the experimental use.

To differentiate MEFs, confluent cells were treated with 20 nM insulin, 0.5 mM dexa-methasone, 0.5 mM 3-isobutyl-1-methylxanthine (IBMX) and 0.125 mM indomethacin for 48 hr. The cells were then switched to medium with insulin only for 6 more days. The differentiation assay for brown pre-adipocytes was similar to that of MEFs except for addition of 1 nM 3,3′,5-Triiodo-L-Thyronine (T3).

### Glucose Tolerance Test (GTT) and Insulin Tolerance Test (ITT)

Glucose-tolerance and insulin-tolerance tests were performed on animals that had been fasted for 16 hours. Animals were injected intraperitoneally with either 1 mg/g body weight of glucose or 0.5 U/Kg body weight of bovine insulin (I5500, Sigma-Aldrich, MO). Glucose levels were determined in blood collected from the tail tip immediately before and 30, 60, 90 and 120 min after the injection using an OneTouch Ultra mini blood glucose monitor (LifeScan, Milpitas, California).

### Measurements of Plasma Parameters

Three-month-old mice were fasted overnight, and blood samples were collected by retro-orbital bleeding. After removal of cells, plasma samples were stored at −80°C for future use. Total cholesterol, HDL-cholesterol, LDL− cholesterol, and triglycerides were determined with reagents from JAS Diagnostics (cholesterol, Cat#CHO2-125; HDL, Cat# HDL3-30; LDL, Cat# LDL3-30 and triglycerides, Cat#TRI2-125) and results were read by “Roche, Cobas Mira” from GMI (Bellevue WA).

### Western Blot Analysis

Epididymal fat tissue was dissected from mice, minced and lysed in T-PER tissue protein extraction buffer (Pierce, Rockford, IL) supplemented with 1 nM phenylmethylsulfonyl fluoride (PMSF) and protease inhibitor cocktail (Roche Diagnostics, Indianapolis, IN). Extracted proteins were separated on 4–20% SDS-PAGE, transferred to cellulose membrane, and probed with appropriate antibodies. Rabbit anti-NG2 antibodies were made in our laboratory [22]. HRP-conjugated secondary antibodies were obtained from Santa Cruz Biotechnology (Santa Cruz, CA).

### Quantitative RT-PCR Analysis

Total RNA was isolated with RNeasy® Lipid Tissue Mini Kit or RNeasy®Mini Kit (Qiagen, La Jolla, CA) following the manufacturer's instructions. Complementary DNA was prepared from 1–2.5 µg of total RNA using the Superscript® First-Strand RT-PCR kit (Invitrogen). Suitable diluted cDNA was used in a 20 µl PCR reaction with Brilliant® II SYBR® Green QPCR Master Mix (Stratagene, La Jolla, CA) and primers at a concentration of 200 nM each. PCR reactions were run in duplicate for each sample and quantified in the MXP 3000 QPCR System (Stratagene, La Jolla, CA). Samples were normalized to acidic ribosomal protein 36B4 mRNA [33] and the expression levels for each test gene were set relative to the average value in the control group, which was defined as 1. Sequences of mouse primers used in this study are: 36B4 forward, 5′-AGC GCG TCC TGG CAT TGT GTG G-3′; 36B4 reverse, 5′-GGG CAG CAG TGG TGG CAG CAG C-3′;TNFα forward, 5′-CCC TCA CAC TCA GAT CAT CTT CT-3′; TNFα reverse, 5′-GCT ACG ACG TGG GCT ACA G; IFNγ forward, 5′- TGC TGA TGG GAG GAG ATG TCT-3′; IFNγ reverse, 5′-TTT CTT TCA GGG ACA GCC TGT T-3′; Il6 forward, 5′-AGA CAA AGC CAG AGT CCT TCA GAG A-3′; Il6 reverse, 5′-GCC ACT CCT TCT GTG ACT CCA GC-3′; Emr-1 forward, 5′-ACA GCC ACG GGG CTA TGG GA-3′; Emr-1 reverse, 5′-GCA CCC AGG AGC AGC CCA AG-3′; β3AR forward, 5′-CGC GTC CGT TTC CCA CGT GA-3; β3AR reverse, 5′-CCA GGG CCG TCA AGC ACA GG-3′;UCP1 forward, 5′- TGC CTG CGG GCA TTC AGA GG-3′; UCP1 reverse, 5-′CCC ATG CAG ATG GCT CTG GGC-3′; UCP2 forward, 5′-CTA CTG TCA GTT CCG CCC TCG GTG T-3′;UCP2 reverse, 5′-AGA GGC TGC GTG GAC CCT CAG-3′; PGC1α forward, 5′-CCA GAG TCA CCA AAT GAC CCC AAG G; PGC1α reverse, 5′-AGC CGG AGA CTG GGC CGT TTA; PRDM16 forward, 5′-CGA ACA CGA GGG CGC ACC A-3′; PRDM16 reverse, 5′-GGC GTC GGC TCC AAA GCT AAC A3′.

### Statistics

Statistical differences between groups were analyzed using the unpaired Student's t test or one-way analysis of variance (ANOVA) with Tukey post-testing using GraphPad Prism 5.0 software. Results are presented as Mean ± SEM. P<0.05 was considered significant.

## Results

### NG2 Ablation Impairs Adipogenesis

**Figure 1:**
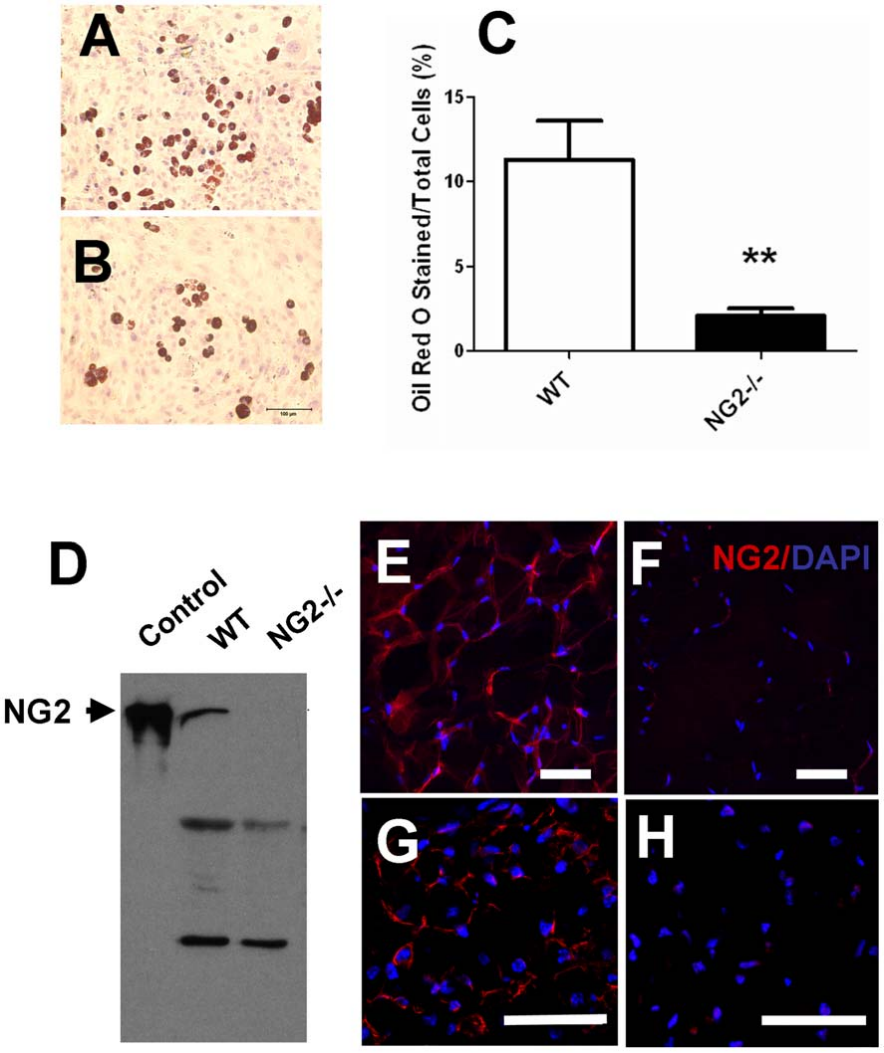
MEF Adipogenesis and Expression of NG2 in Adipocytes. (A and B) Oil red O staining to visualize differentiated adipocytes from wild-type (A) and NG2 null (B) MEFs. (C) Percentages of Oil red O stained cells *vs* total cells were determined in 10 randomly-selected high power fields. Results are quantified as Mean ± SEM. **, P<0.01. (D) Western blot analysis of NG2 protein in extracts of epididymal fat tissue from 3 month old wild-type and NG2 null mice. (E and F) Immunofluorescence labeling of NG2 in 20 µm-thick cryosections of epididymal fat tissue from 3-month old wild-type and NG2 null mice. (G and H) Immunofluorescence labeling of NG2 in 10 µm-thick cryosections of interscapular brown fat tissue from postnatal day 5 wild-type and NG2 null mice. Bar = 50 µm.

Our laboratory has previously reported that the NG2 proteoglycan is expressed by white adipocytes in the subcutis layer of early postnatal mouse skin. Furthermore, we found that genetic ablation of NG2 leads to deficits in adipocyte development and skin thickness [20], suggesting that NG2 promotes adipogenesis in newborn mice. To follow up on these observations, we isolated murine embryonic fibroblasts (MEFs) from both wild-type and NG2 null E13.5 embryos and cultured them under conditions suitable for inducing adipogenesis (see Methods). Staining these cultures after eight days with Oil red O to visualize differentiated adipocytes revealed a large decrease in adipogenesis in NG2 null MEFs compared to wild-type MEFs, supporting the concept that NG2 plays a role in adipogenesis (Fig. 1 A–C).

### NG2 Null Mice Develop Mature-Onset Obesity

**Figure 2:**
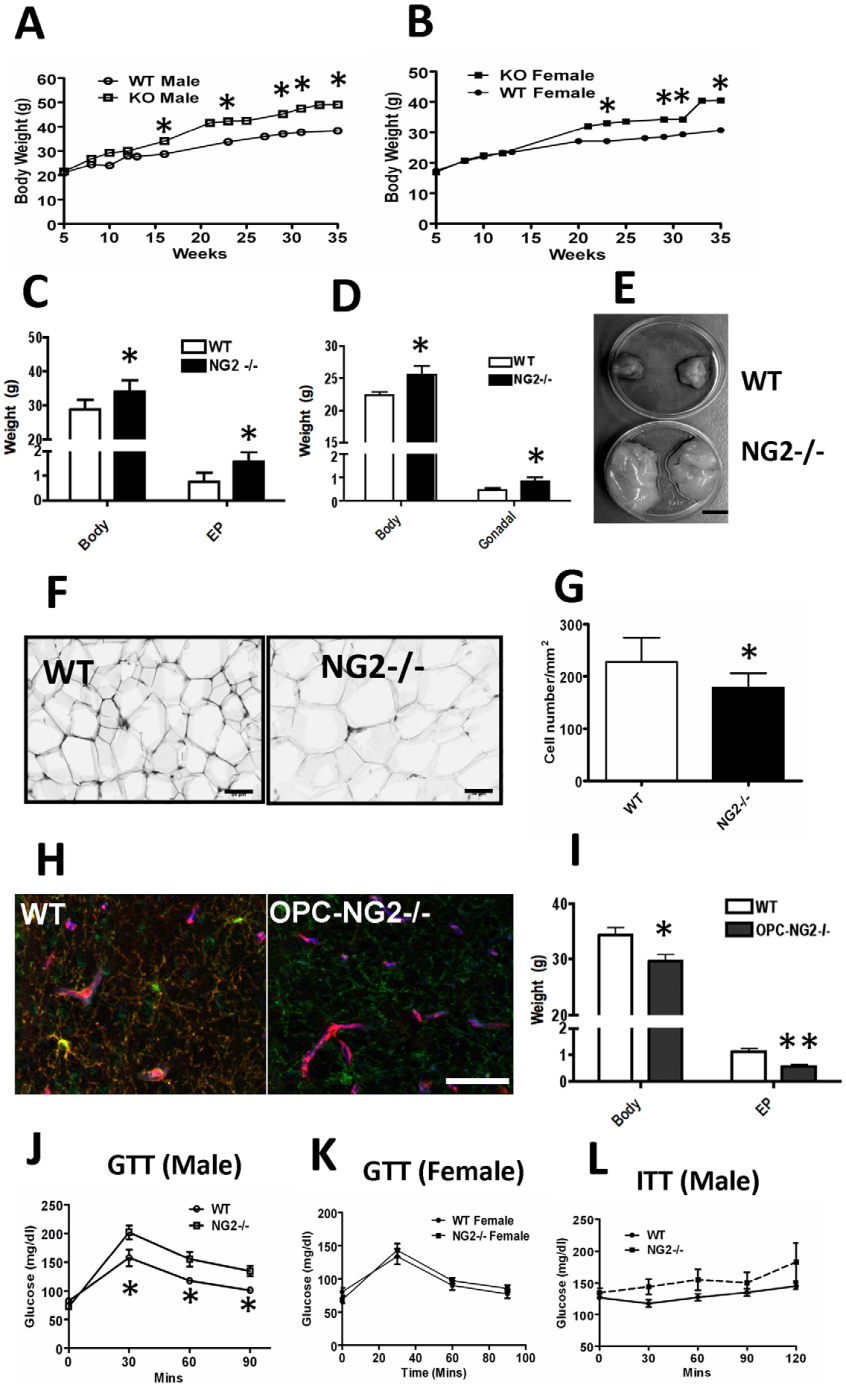
NG2 Null Mice are obese. (A and B) Body weight gain in wild-type and NG2 null mice. (C) Body weight and epididymal fat weight (grams) of 16-week old male wild-type and NG2 null mice (n = 6–10). (D) Body weight and weight of gonadal fat deposits in 16 week female mice (n = 7–9). (E) Dissected epididymal fat pads from 16-week old male wild-type and NG2 null mice. Fat pads are shown in 35-mm dishes. (F) Sizes of white fat cells in sections of epididymal fat pads from 16-week old male wild-type and NG2 null mice. Bar = 50 µm. (G) Numbers of fat cells per mm^2^ (n = 5). (H) Expression of NG2 (Red), PDGFRα(Green) and CD31 (Blue) in sections through the ventromedial hypothalamic nucleus in both wild-type and OPC-NG2 Knockout mice. Note that yellow color in WT is due to red/green overlap. NG2 is not expressed by OPCs in the OPC-NG2 null mouse. Bar = 50 µm. (I) Body weight and epididymal fat weight in 16-week old wild-type and OPC-NG2 null mice (n = 8–9). (J and K) GTT (at 16 weeks) in male and Female wild-type and NG2 null mice (n = 5). (L) ITT (at 25 weeks) in male wild-type and NG2 null mice (n = 5). *, p<0.05; **, p<0.01.

Shifting our focus to other adipose deposits, we confirmed that NG2 is expressed by both white adipocytes in epididymal fat pads and brown adipocytes in interscapular BAT (Fig. 1 D–H). As an initial means of determining whether NG2 ablation affects adipogenesis in adult mice, we compared total body weights of wild-type and NG2 null mice (on normal chow) from the ages of 1–8 months. In contrast to our experience with early postnatal mice, between 3–4 months of age, NG2 null mice began to exhibit more weight gain than wild-type counterparts (Fig. 2 A and B), maintaining a 20% weight surplus throughout adulthood. Fig. 2 C illustrates this statistically significant weight difference for male mice at the age of 16 weeks. Strikingly, the size of epididymal fat deposits in NG2 null male mice was visibly larger than that seen in wild-type male mice (Fig. 2 E), exhibiting a two-fold increase in weight at the age of 16 weeks (Fig. 2 C). Increased body weight and increased sizes of gonadal fat deposits were also observed in NG2 null female mice (Fig. 2 D). H & E staining of sections of epididymal fat pads revealed that the size of adipocytes in NG2 null mice is 20% larger than that seen in wild-type mice (Fig. 2 F). Wild-type tissue contains about 220 fat cells per mm^2^ versus only 180 fat cells per mm^2^ in NG2 null fat (Fig. 2 G). These data indicate NG2 null mice may become obese due to increased lipid accumulation by NG2 null white adipocytes. We are thus faced with the paradoxical finding that NG2 null mice exhibit mature-onset obesity in spite of deficits in the development of white adipose tissue observed during early postnatal life.

### CNS Dysfunction is Not Responsible for Obesity in the NG2 Null Mouse

Because signaling from the hypothalamus plays a large role in sympathetic nervous system control of brown fat function, we considered whether ablation of NG2 in the central nervous system (CNS) might be responsible for the obese phenotype of the NG2 null mouse. In the CNS, NG2 is expressed by oligodendrocyte progenitor cells (OPCs) that give rise to myelinating oligodendrocytes. Since we have shown that ablation of NG2 results in myelination deficits due to reduced OPC proliferation [28], it is possible that hypomyelination might result in reduced signaling from the hypothalamus to the sympathetic neurons that control brown fat activity. To check this possibility, we crossed NG2 floxed mice with Olig2-Cre mice to generate an OPC-specific NG2 (OPC-NG2) null mouse (Materials and Methods). Immunolabeling demonstrated that NG2 was expressed by both microvascular pericytes and OPCs in the ventromedial hypothalamic nucleus of control (flox/flox) mice. However, in OPC-NG2 null mice, NG2 was expressed only by pericytes (Fig. 2 H), demonstrating the specificity of NG2 ablation in OPCs. Contrary to the situation in global NG2 null mice, OPC-NG2 null mice are leaner than control mice (Fig. 2 I). Moreover, the weights of epidydimal fat deposits in OPC-NG2 null mice are reduced 2-fold compared to control mice (Fig. 2 I). These observations strongly suggest that obesity in the global NG2 null mouse does not arise from NG2 ablation in the CNS.

### Glucose and Insulin Tolerance are Impaired in NG2 Null Mice

Because of the frequent correlation of obesity with diabetes, we wondered if adult NG2 null mice exhibited abnormalities in glucose regulation. Accordingly, glucose tolerance testing (GTT) was performed in 16-week old male mice. Data shown in Fig. 2 J suggests that male NG2 null mice exhibit a small but statistically significant glucose intolerance compared to wild-type males. Glucose tolerance appeared normal in 16-week old female NG2 null mice (Fig. 2 K). Similarly, a trend toward insulin intolerance was found in 25-week old male NG2 null mice compared to wild-types, although this difference was not statistically significant (Fig. 2 L). These data indicate that male NG2 null mice exhibit mild pre-diabetic symptoms as they age.

### NG2 Null Mice Develop Liver Steatosis

**Figure 3:**
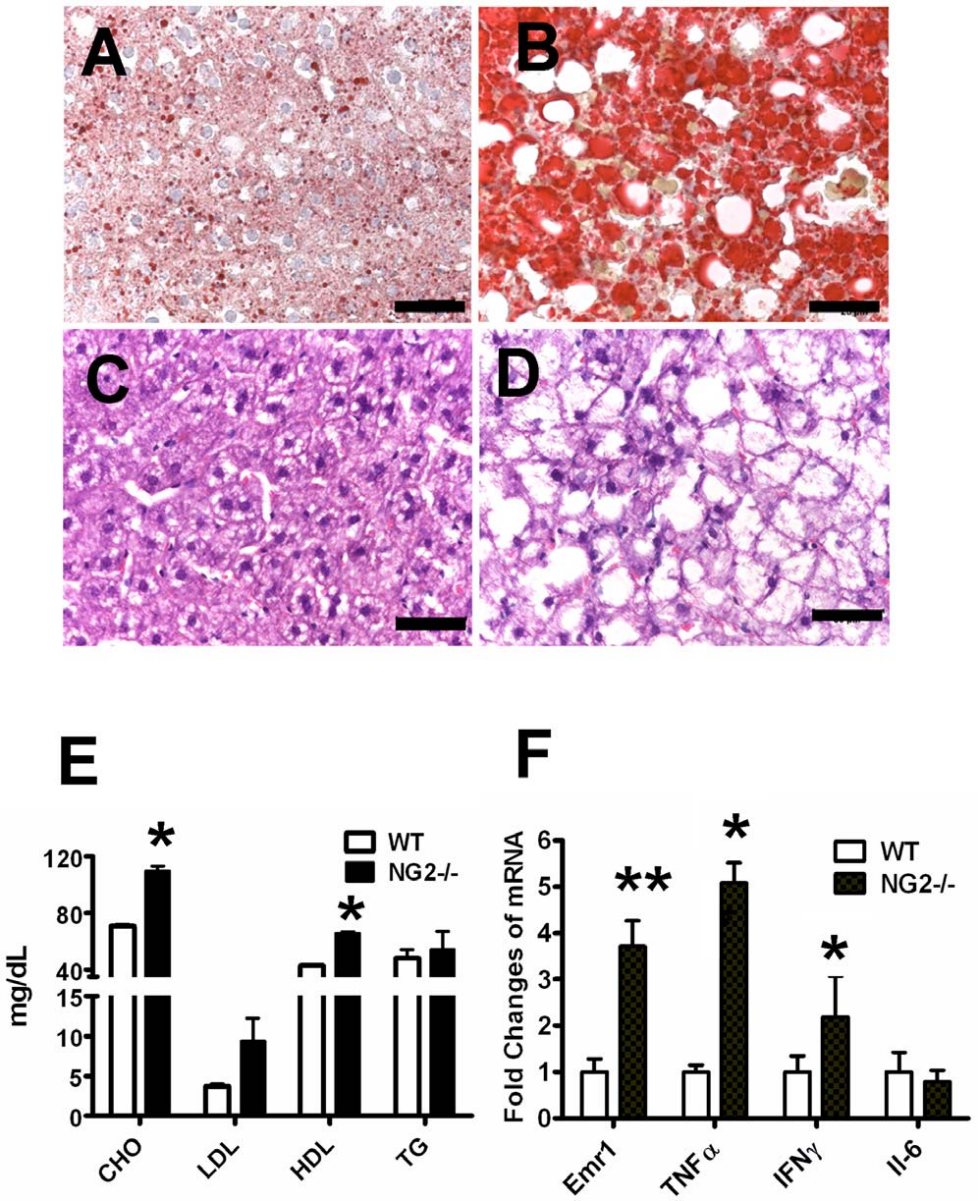
Obesity-associated alterations in NG2 Null Mice. (A and B) Oil Red O staining of liver sections from 10-month old male wild-type (A) and NG2 null (B) mice. (C and D) H & E staining of liver sections from 10-month old male wild-type (C) and NG2 null (D) mice. Bar = 50 µm. (E) Plasma lipid levels in 10-month old male wild-type and NG2 null mice (n = 10). (F) Messenger RNA levels for several myeloid-related cytokines in epididymal fat from 10-month old male wild-type and NG2 null mice (n = 3). *, P<0.05; **, P<0.01.

Male NG2 null mice also exhibit fatty livers as they age. Sections of livers from 10-month old mice were stained by Oil red O and H & E. Fig. 3 shows that liver sections from male NG2 null mice are much more heavily stained by Oil red O than sections from wild-type males (Fig. 3 A and B), indicative of excessive lipid accumulation by hepatocytes in NG2 null mice. This NG2 null phenotype is also evidenced in H & E sections by the presence of numerous cleared areas caused by xylene extraction of lipid during the staining process (Fig. 3 C and D). A possible underlying cause of excessive lipid storage in hepatocytes is suggested by elevated levels of total cholesterol, HDL-cholesterol, and LDL− cholesterol in the plasma of NG2 null male mice compared to wild-type mice (Fig. 3 E).

### Macrophages are Activated in NG2 Null Mice

Since macrophage activation in WAT is often correlated with obesity [34], [35], we quantified mRNA levels of several myeloid-related cytokines in epididymal fat tissue from 10-month old male mice. Our quantitative PCR data show that message levels for both TNFα (6-fold) and IFNγ (2-fold) are significantly increased in WAT of NG2 null mice compared to wild-type mice. Message levels of Emr1 (F4/80), a well-known macrophage marker, are also increased about 4-fold in NG2 null mice (Fig. 3 F). These results are consistent with the idea that macrophages are activated toward an M1 inflammatory phenotype in WAT from NG2 null mice.

### Decreased Energy Expenditure in NG2 Null Mice

**Figure 4:**
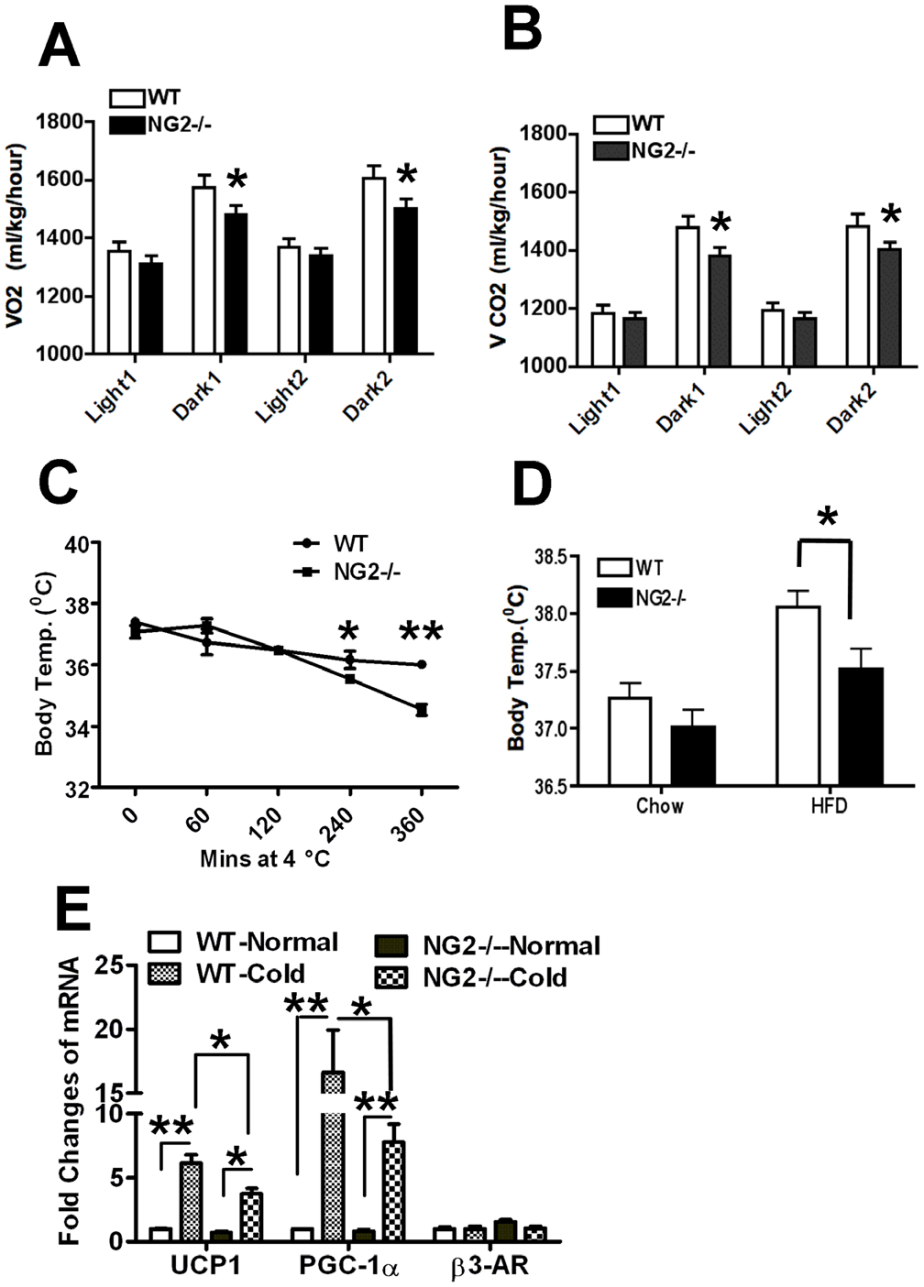
Metabolic and Cold Challenge Testing in Wild-type and NG2 Null Mice. (A and B) Volume of O_2_ consumption (A) and CO_2_ production (B) by 3-month old wild-type and NG2 null mice over 48 hours (Two light/dark cycles, n = 15). Data were normalized to body weights of individual mice. (C) Cold challenge testing of 3-month old male wild-type and NG2 null mice (n = 5). (D) Three month old male wild-type and NG2 null mice were put on HFD for 10 weeks and body temperatures were measured. As a control, body temperatures of five month old male wild-type and NG2 null mice on regular chow were measured (n = 6–7). (E) Levels of mRNA for brown fat-related genes in IBAT of wild-type and NG2 null mice maintained at room temperature or at 4°C for 6 hours (n = 3). *, P<0.05; **, P<0.01.

Obesity can result from an imbalance between calorie intake and energy expenditure. To evaluate this possibility in the case of NG2 null mice, we performed metabolic testing on 3-month old wild-type and NG2 null mice. These tests revealed no significant change in food intake or activity in NG2 null mice when compared to wild-type mice. However, relative to wild-type mice, NG2 null mice exhibit decreases in both oxygen consumption and carbon dioxide production, especially during the dark cycle when activity levels are highest (Fig. 4 A and B). These data indicate a possible decrease in energy expenditure or utilization in NG2 null mice. Significantly, these results were obtained at 13 weeks of age, prior to the divergence of body weights seen in Fig. 2A and B. NG2-dependent metabolic deficits are therefore likely to be the cause rather than the consequence of obesity.

### Adaptive Thermogenesis is impaired in NG2 Null Mice

Defective energy utilization is often indicative of impaired brown fat function. For an initial assessment of brown fat function in NG2 null mice, we examined adaptive thermogenesis, defined as heat production in response to environmental temperature or diet [36]. We performed a cold challenge test in which 3-month old male wild-type and NG2 null mice were kept at 4°C for up to 6 hours, with hourly measurements of body (rectal) temperature. Compared to wild-type mice, NG2 null mice failed to maintain their body temperature after 4 hours of cold exposure. After 6 hours in the cold, we found a 1.5 degree difference between the body temperatures of wild-type and NG2 null mice (Fig. 4 C). Similarly, at room temperature we found that body temperatures of NG2 null mice maintained on a high fat diet (HFD) were 0.5 degree lower than those of wild-type mice on HFD. On regular chow, body temperatures of wild-type and NG2 null mice were not significantly different (Fig. 4 D). Since both the cold challenge and HFD tests are diagnostic of brown fat function, these results indicate abnormalities in the brown fat tissue of NG2 null mice. As in the case of metabolic deficits (Fig. 4A and B), changes in cold sensitivity were observed at 13 weeks, prior to the onset of excess weight gain in the NG2 null mice.

For more direct evidence concerning brown fat function, we used real time Q-RT-PCR analysis of interscapular brown fat tissue (IBAT) to quantify mRNA levels for genes related to brown fat function. We found that mRNA levels for both UCP1 and PGC1-α were dramatically increased in wild-type mice after a 6-hour cold exposure, consistent with published data on brown fat activity in response to cold challenge [37], [38]. UCP1 and PGC1-α message levels were also increased in NG2 null mice following cold challenge, but to a much lesser degree than in wild types (Fig. 4 E), suggesting an underlying mechanism for the inability of the mutant mice to maintain body temperature. No significant changes were found in message RNA levels for β3 adrenergic receptor (Fig. 4 E), which is an upstream regulator of PGC-1α [6]. Together, the adaptive thermogenesis and gene expression data are strongly suggestive of impaired brown fat function in NG2 null mice.

### Development of IBAT is Impaired in Newborn NG2 Null Mice

**Figure 5:**
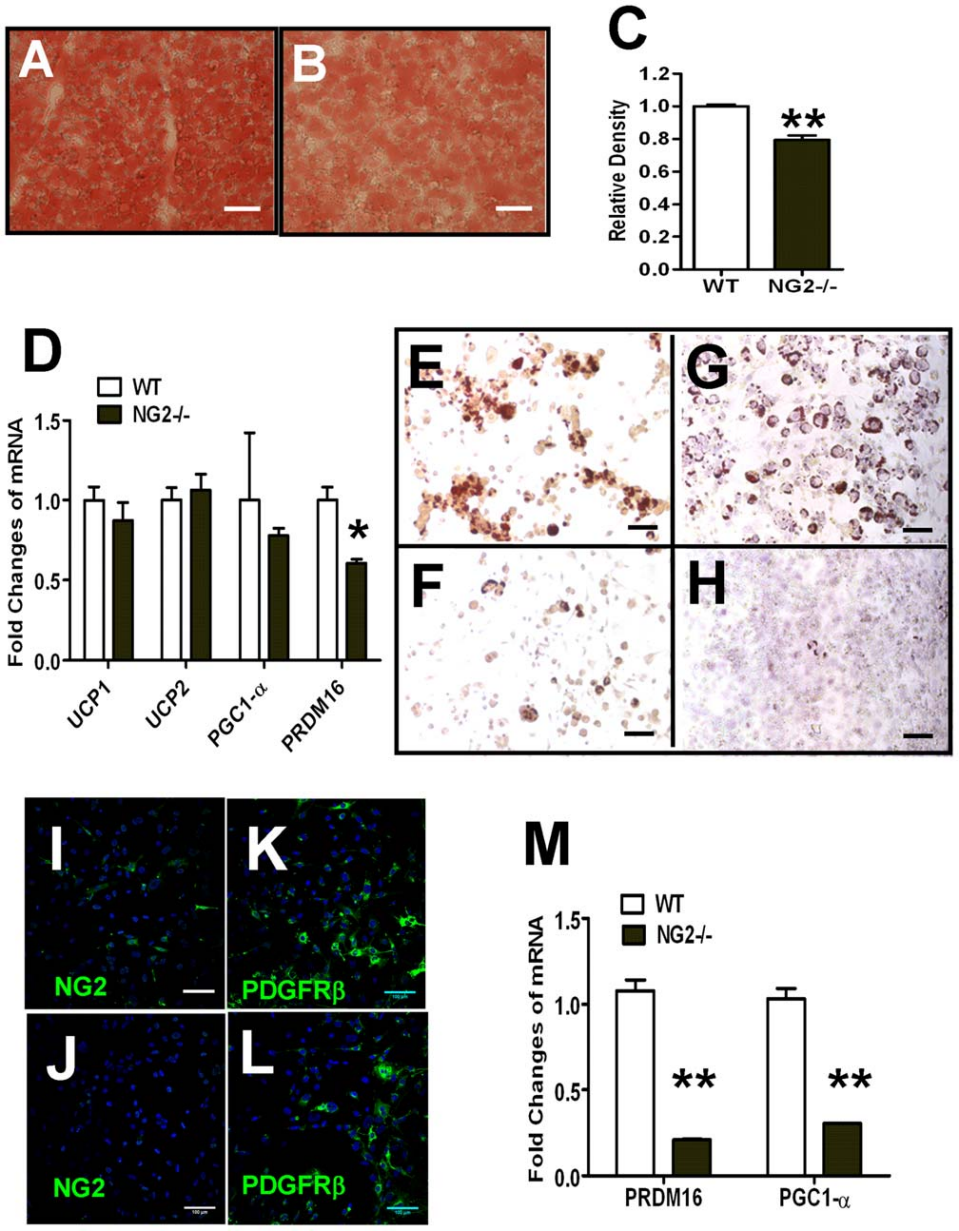
Brown Fat Development in Wild-type and NG2 Null Mice. (A–C) Oil Red O staining of IBAT from P5 wild-type (A) and NG2 null (B) mice and quantification of Oil Red O staining intensity (C, n = 6). Bar = 50 µm. D. mRNA levels for brown fat-related genes in IBAT from P5 wild-type and NG2 null mice (n = 3). (E–H) Oil Red O staining of primary wild-type (E) and NG2 null (F) as well as immortalized wild-type (G) and NG2 null (H) pre-brown fat cells after 5 days of differentiation. Bar = 50 µm. (I–L) Immuno-fluorescence to detect NG2 and PDGFRβ in immortalized wild-type brown pre-adipocytes (I and K) and NG2 null brown pre-adipocytes (J and L). Bar = 100 µm. (M) Messenger RNA levels of PRDM16 and PGC1-α in immortalized pre-brown fat cells from wild-type and NG2 null mice (n = 3). *, P<0.05; **, P<0.01.

Since NG2 is frequently expressed by progenitor cells, including mesenchymal stem cells [39] and is also expressed in mature brown adipocytes (Fig. 1 G and H), we wondered whether NG2 ablation causes defects in brown pre-adipocytes that are responsible for abnormal development of brown fat tissue. First, we checked the weight and morphology of IBAT in mice at postnatal day 5 (P5), and at the ages of one and three months. We found no obvious difference in the weight (ratio of weight of IBAT *vs.* body weight) or color of wild-type *vs.* NG2 null IBAT. However, Oil red O staining in P5 IBAT sections was reduced in NG2 null mice compared to wild-type mice (Fig. 5 A–C), consistent with our previous finding of reduced lipid content in white adipocytes in early postnatal NG2 null skin [20]. Furthermore, Q-RT-PCR assays showed that mRNA levels for PRDM16 (p<0.05) and PGC1-α were reduced in IBAT from NG2 null mice, although the latter difference was not statistically significant (Fig. 5 D). These data are indicative of a deficit in the functional properties of BAT in NG2 null mice, even though overall IBAT mass is unchanged.

To further evaluate brown fat development, we isolated and expanded primary brown pre-adipocytes from IBAT of newborn mice (P1) and studied the induction of adipogenesis in vitro. After eight days of differentiation, we found that Oil red O stained cells were infrequently seen in primary cultures of NG2 null brown pre-adipocytes compared to their wild-type counterparts (Fig. 5 E and F), suggesting that brown adipogenesis is impaired in the absence of NG2 proteoglycan. We also immortalized brown pre-adipocytes via SV40 virus infection as described in Methods. Immuno-staining revealed that NG2 is highly expressed in about 30% of immortalized wild-type pre-brown fat cells, but is not present in immortalized NG2 null pre-brown fat cells. The expression of PDGFRβ, another mesenchymal stem cell marker, was similar in both wild-type and NG2 null immortalized pre-brown fat cells (Fig. 5 I–L). As seen with primary brown pre-adipocytes, adipogenesis was dramatically reduced in immortalized NG2 null brown pre-adipocytes compared to wild-type cells, as judged by Oil Red O staining (Fig. 5 G and H). These data indicate that NG2 ablation in pre-brown fat cells results in a reduced capacity for differentiation to mature brown adipocytes.

We also used real time RT-PCR to measure mRNA levels for PRDM16 and PGC1-α in immortalized pre-brown fat cells. Message RNA levels for both PRDM16 and PGC1-α were dramatically decreased in immortalized NG2 null pre-brown fat cells compared to wild-type cells (Fig. 5 M), suggesting deficits in PRDM16-mediated signaling as a basis for impaired brown adipogenesis in the absence of NG2 proteoglycan. It is important to note that these deficits in brown adipocyte function, occurring both in vivo and in vitro, are observed during the first week postnatally, well before increased weight gain in the NG2 null mouse. This again is consistent with the theme that brown fat dysfunction in the NG2 null mouse is not the consequence of obesity, but instead might provide a basis for excess weight gain in adult NG2 null mice.

Taken altogether, these findings support the conclusion that NG2 ablation impairs development of brown adipose tissue in much the same way that we previously observed for white adipocytes in postnatal skin [20]. The resulting brown fat deficit is responsible for poor energy utilization/expenditure that leads to storage of excess lipid in white adipocytes and hepatocytes. The obese/fatty liver phenotype of the NG2 null mouse is thus due to an indirect effect of deficient brown fat function on white adipocytes and hepatocytes.

## Discussion

A number of pieces of *in vitro* data suggest that the NG2 proteoglycan promotes the proliferation and motility/recruitment of immature cells and tumor cells, acting via potentiation of signaling through β1 integrins and growth factor receptors [25]. This function of NG2 is borne out by *in vivo* studies on the NG2 null mouse that reveal deficits in the development of cell types that are normally positive for NG2. Studies of pathological microvessels in the NG2 null mouse demonstrate reduced recruitment of pericytes and reduced pericyte ensheathment of endothelial cells [26], [27]. In the central nervous system, oligodendrocyte progenitors in the NG2 null mouse do not proliferate normally, resulting in production of reduced numbers of mature oligodendrocytes and deficits in myelination [28]. In early postnatal skin, generation of both keratinocytes and white adipocytes is sub-normal in the NG2 null mouse, leading to transient deficits in skin development [20].

In light of these developmental deficits, it has been surprising to discover that NG2 null mice develop adult-onset obesity, with adult males and females weighing as much as 20% more than wild-type counterparts due to white adipose deposits that can be up to twice as large as corresponding wild-type deposits. The increased fat pad size is due at least in part to a 20% increase in the diameter of white fat cells, indicating an increase in lipid storage in NG2 null mice. This increased storage may be linked to increased serum lipid levels in the NG2 null mouse, a factor that may also explain the occurrence of severe liver steatosis in older males. Male NG2 null mice also exhibit mild diabetic symptoms, including glucose intolerance and insulin resistance. The hypertrophy of white adipose tissue in NG2 null mice stands in vivid contrast to the hypotrophic phenotypes of other tissues studied in these mice, including early postnatal white adipose tissue [20]. This paradox suggested the possibility that the unexpected effect of NG2 ablation on white fat might be an indirect one caused by NG2 ablation in some other cell type.

One scenario was that NG2 ablation in OPCs in the CNS might affect hypothalamic control of metabolism due to deficits in neuronal impulse conduction resulting from hypomyelination of axons. This possibility was ruled out by the finding that OPC-specific ablation of NG2 yielded a phenotype opposite to that seen in the global NG2 null mouse; namely, the OPC-specific NG2 null mice were leaner than control mice with two-fold smaller epididymal fat pads. An alternative clue regarding the basis of the global NG2 null phenotype was provided by metabolic studies that revealed decreased production of CO_2_ and decreased consumption of O_2_ in NG2 null mice, in spite of food intake and activity levels that matched those of wild-type mice. These findings suggested an alteration in the balance between energy expenditure and energy storage in NG2 null mice. Since energy homeostasis depends heavily on the function of brown adipose tissue, we looked for evidence of impaired brown fat function in NG2 null mice. Indirect evidence for such a defect was seen in the performance of NG2 null mice when challenged with cold exposure or high fat diet feeding. In both types of tests, NG2 null mice failed to maintain normal body temperature, diagnostic of impaired adaptive thermogenesis, a process that depends on efficient brown fat function.

More direct evidence for deficits in brown fat function in the cold challenge test was obtained by examining levels of transcripts for the uncoupling protein UCP1 and the cold-inducible transcriptional activator PGC1-α, both of which are normally highly up-regulated in response to cold exposure [38]. Up-regulation of both UCP1 and PGC1-α were reduced in NG2 null mice following cold exposure. Changes in levels of transcripts for the β3 adrenergic receptor were not affected by NG2 ablation, showing that effects of NG2 ablation on UCP1 and PGC1-α are not due to NG2-dependent alterations in adrenergic receptor levels. From a developmental standpoint, interscapular brown fat from postnatal day 5 NG2 null mice exhibited reduced lipid content, although BAT mass was similar to wild-type controls. More importantly, NG2 null IBAT exhibited reduced levels of transcripts for both PGC1-α and PRDM16, factors that are required for brown fat development [3], [4], [40]. In parallel, we were able to demonstrate a large deficit in the ability of NG2 null brown pre-adipocytes to undergo differentiation in cell culture. As seen in vivo, NG2 null brown pre-adipocytes also exhibited great reduced levels of PGC1-α and PRDM16.

A recurring theme in our data is that various deficits associated with brown fat function occur prior to the time at which NG2 null mice begin to exhibit excess weight gain. This is especially true of the adipogenesis assays performed with brown adipocytes isolated from postnatal day 5 mice and of the PGC1-α and PRDM16 transcription assays performed with both whole tissue and cultured adipocytes at this same early time point. It is also true of the metabolic and cold challenge tests performed with 13-week old mice. Taken together, these findings provide a firm basis for concluding that excess weight gain is unlikely to be responsible for the brown fat dysfunction observed in NG2 null mice. Instead, it is much more likely that impaired brown fat development and/or function underlies the obese phenotype of the NG2 null mouse. Impaired energy expenditure in the NG2 null mouse likely leads to increased storage of excess lipids by white adipocytes, resulting in the observed increase in adipocyte size and fat pad size. This NG2-dependent deficit in brown fat development is more in line with our general “rule” that NG2 is normally involved in expansion of immature cell populations and that its ablation results in subnormal development. In this case, deficits in BAT development/function have indirect hypertrophic effects on white adipocytes that appear to override the direct early hypotrophic effect of NG2 ablation on this population. In light of recent evidence concerning the importance of BAT in adult humans, this interplay between BAT and WAT is of significant interest.

Additional work is required to understand the mechanism by which NG2 ablation affects both adaptive thermogenesis and brown adipocyte development. Both processes are dependent on activation of β3 adrenergic receptor, leading to cAMP-mediated activation of protein kinase A (PKA) [6]. In the former case, PKA activation stimulates lipolysis and UCP1 activation to produce heat. In the latter case, PKA activation mediates phosphorylation of the cAMP responsive element binding protein (CREB) to induce differentiation of brown pre-adipocytes [6]. It is therefore tempting to speculate that, as a membrane-spanning protein, NG2 could be involved in potentiating the activity of either the G protein-coupled β3 adrenergic receptor or adenylate cyclase itself. However, no evidence currently exists for involvement of the proteoglycan in adrenergic signaling. More realistic possibilities are based on our findings that NG2 promotes activation of signaling through β1 integrins [41], [42], [43], [44] and through growth factor receptors for FGF and PDGF [17], [29], [45]. While neither integrins nor growth factor receptors represent traditional G protein-coupled receptors, there are nevertheless numerous reports of G protein-dependent and G protein-independent activation of PKA by β1 integrin [46], [47], [48] and receptors for FGF or PDGF [47], [49], [50], [51]. Future work will examine the ability of NG2 to activate PKA signaling via β1 integrin and growth factor receptor-dependent mechanisms. New research efforts will also be directed toward understanding the basis of the lean phenotype of the OPC-specific NG2 null mouse. NG2 clearly has the potential to affect metabolism via its actions in several different tissues, making the proteoglycan a subject of substantial interest in the field of metabolism.
